# Haplotype analysis of the Apolipoprotein A5 gene in Moroccan patients with the metabolic syndrome

**DOI:** 10.1186/s40200-015-0160-3

**Published:** 2015-04-16

**Authors:** Maria Ajjemami, Sanaa Ouatou, Hicham Charoute, Malika Fakiri, Houria Rhaissi, Houda Benrahma, Hassan Rouba, Abdelhamid Barakat

**Affiliations:** Département de Recherche Scientifique, Laboratoire de Génétique Moléculaire Humaine, Institut Pasteur du Maroc, 1, Place Louis Pasteur, 20360 Casablanca, Morocco; Univ Hassan 1, Laboratoire Agroalimentaire et Santé, 26000 Settat, Morocco; Laboratoire de Physiologie et Génétique Moléculaire, Faculté des sciences Ben M’Sik, Université Hassan II, Mohammedia, Morocco

**Keywords:** Metabolic Syndrome, *APOA5* gene, Polymorphisms, Haplotypes

## Abstract

**Background:**

In this case–control study we investigated the relative contribution of commons APOA5 polymorphisms and haplotypes to the risk of metabolic syndrome in Moroccan patients.

**Methods:**

Using the International Diabetes Federation (IDF) criteria for metabolic syndrome, the study included 176 patients and 105 controls. We genotyped APOA5 polymorphisms (−1131 T > C, c.56C > G, c.553G > T and c.1259 T > C) by PCR-RFLP analysis. The effects of APOA5 polymorphisms and constructed haplotypes on metabolic syndrome were estimated using logistic regression analyses.

**Results:**

The statistical analysis showed a significant association between APOA5 -1131 T > C and APOA5 c.56C > G polymorphisms with metabolic syndrome in both Codominant and Dominant models. The APOA5 -1131 T > C polymorphism was associated with increased fasting glucose (p = 0.0295) and reduced HDL levels (p = 0.0091). Carriers of the APOA5 c.56G allele had increased triglyceride levels (p = 0.0435) and waist circumference (p = 0.0122). Similarly the APOA5 1259 T > C variant was associated with increased waist circumference (p = 0.0463). The haplotypes CCGT (OR = 3.223; p = 0.00278) and CGGT (OR = 8.234; p = 0.00534) were significantly associated with susceptibility to metabolic syndrome.

**Conclusions:**

Our results confirms the association of APOA5 -1131 T > C and c.56C > G variants with the predisposition to metabolic syndrome complications.

## Background

The metabolic syndrome (MS) is characterized by the clustering of several factors: central obesity, raised blood pressure, raised fasting glucose, elevated triglycerides (TG) and reduced high-density lipoprotein (HDL) cholesterol [1]. Hypertriglyceridemia is the essential lipid abnormality in MS, and is a direct consequence of the visceral accumulation of fat, for which the waist circumference is the best surrogate marker [2].

In urban Moroccan women, the prevalence of MS was about 17.8% [3]. It is a multifactorial disease, besides environmental factors such as cigarette smoking, obesity, lack of exercise and bad nutrition habits, genetic factors also contribute to its pathogenicity [4,5]. Apolipoprotein A5 gene (APOA5) was identified 30 kb upstream of the well-characterized APOA1/C3/A4 gene cluster on chromosome 11. Several studies suggested a strong correlation between APOA5 and plasma triglycerides levels [6]. Human APOA5 gene consists of four exons and encodes a 366-amino acid protein, which is produced only by the liver and associated with high (HDL) and very low (VLDL) density lipoproteins particles [7]. Numerous studies have confirmed the associated between APOA5 alleles such as 1131C, IVS3 + 476A, 1259C, 56G and elevated triglyceride levels [8,9]. In addition, some APOA5 polymorphisms confer increased risk for the development of coronary heart disease and ischemic stroke [10,11].

Two APOA5 common polymorphisms; the −1131 T > C (SNP3) in the promoter region, and the c.56C > G in the exon 3 were significantly associated with TG levels in several population [12-16]. The APOA5 gene variant, c.553G > T (substitution of a cysteine for a glycine residue at amino acid residue 185(G185C)) was associated with higher TG levels in Asian populations [17-19]. In contrast, this variant was rare or absent in Caucasian populations [13,20].

The goal of the present study was to assess the relative contribution of SNPs in the APOA5 gene to the risk of metabolic syndrome and we studied the major APOA5 haplogroup profiles in MS patients in Moroccan Patients.

## Methods

### Study subjects

We dispose 283 subjects recruited from Pasteur Institute of Morocco in Casablanca, aged between 20 and 60 years, are from various geographic regions of the country and are from different ethnic origin (Arab, Amazigh and Sahraouis). Metabolic syndrome was diagnosed according to the criteria of International Diabetes Federation (IDF) [21]. All patients must have central obesity (waist circumference ≥ 94 cm for males or ≥ 80 cm for females) plus at least two of the following criteria: (1) raised triglycerides level: ≥1.7 mmol/l (150 mg/dL) (or specific treatment for this lipid abnormality); (2) reduced HDL-cholesterol: < 1.03 mmol/L (40 mg/dL) in males and < 1.29 mmol/l (50 mg/dl) in females (or specific treatment for these lipid abnormality); (3) raised blood pressure (BP): systolic BP ≥130 or diastolic BP ≥85 mmHg (or treatment of previously diagnosed hypertension); (4) raised fasting plasma glucose (FPG): FPG ≥5.6 mmol/l (100 mg/dl) (or previously diagnosed type 2 diabetes). Subjects who met the IDF definition for metabolic syndrome were defined as patients, and the remaining individuals were assigned to the control group. The exclusion criterion of patients and controls was the presence of any disease known to influence serum lipid levels, such as thyroid disease, renal failure or liver disease. All participants signed informed consent forms, and the study protocol was approved by local Committee on Research Ethics of Pasteur Institut of Morroco.

### Clinical and biochemical measurements

Clinical and biochemical parameters were measured by standard laboratory procedures. Weight and height were measured to determine body mass index (BMI). BMI was calculated from height and weight of the individual by the following formula: Weight (kg)/height (m)^2^ [22]. Height is usually measured to the nearest 0.5 cm with stadiometer, without shoes. The weight is measured to the nearest 0.1 kg on a medical balance scale, the individual is lightly dressed and without shoes Waist and hip circumference were also measured. The systolic and diastolic blood pressure was measured using a sphygmomanometer after 5 minutes minimum of rest in a sitting position. Both strains were expressed in mmHg (millimeters of mercury). At the end of each questionnaire, a blood sample was collected in 2 tubes (for biochemical analysis and for DNA extraction). Fasting glucose, triglyceride (TG), total cholesterol (Total-C), LDL cholesterol (LDL-C) and HDL cholesterol (HDL-C) were measured after 8 hours of fasting. All assays were performed using an automatic (VITROS).

### Molecular analysis

Genomic DNA was extracted from peripheral blood leukocytes by standard phenol-chloroform method. To detect the -1131 T > C (rs662799), c.56C > G (rs3135506), c.553G > T (rs2075291) and c.1259 T > C (rs2266788) polymorphisms, polymerase chain reaction (PCR) conditions and restriction fragment length polymorphism (RFLP) analyses were performed according to previous published protocols [23-26] (Table 1).

**Table 1:** **primers and restriction enzymes used for identification of APOA5 polymorphisms**

*APOA5* SNP	forward (F) and reverse (R) primers	restriction enzymes	PCR-RFLP products	Reference
c.1259 T > C (rs 2266788)	F: 5’- TCA GTC CTT GAA AGT GGC CT-3’ R: 5’- ATG TAG TGG CAC AGG CTT CC -3’ PCR-product: 287 pb	BseGI	TT : 122 and 165 bp	[26]
			CC : 35, 87and 165 bp	
−1131 T > C (rs662799)	F: 5’-CCC CAG GAA CTG GAG CGA AATT-3’ R: 5’-TTC AAG CAG AGG GAA GCC TGT A -3’ PCR-product:396 bp	TruI	TT : 20, 105, 271 bp	[24]
			CC : 105 and 291 bp	
c.56C > G (rs3135506)	F: 5’- GGC TCT TCT TTC AGG TGG GTCTCCG -3’ R:5’- GCC TTT CCG TGC CTG GGT GGT-3’ PCR-product: 157 pb	Taq I	CC : 134 and 23 bp	[23]
			GG : 157 bp	
c.553G > T (rs 2075291)	F:5’-AGA CAC CAA GGC CCA GTT GCT GGG-3’ R: 5’- ATG CCG CTC ACC AGG CTC TCG GCG -3’ PCR-product: 138 pb	HaeIII	TT: 127 and 11 bp	[25]
			GG : 76, 51 and 11 bp	

### Statistical analysis

Quantitative data were expressed as means ± standard deviation (SD). Differences between subject groups, genotypes and haplotypes for continuous variables were assessed by Student test. Manne-Whitney test was used for variables not normally distributed. Chi-square test was applied to examine differences in genotype distributions between cases and controls. Odds ratios (OR) with 95% of confidence intervals (CI) were calculated to assess strength of association. All ORs were adjusted for sexe, age and body mass index (BMI). Statistical analyses were performed using STATA software, version 11.0. The PLINK software v1.07 was used for haplotype frequencies estimation and comparison.

## Results

Clinical and biochemical characteristics of MS patients and control subjects are shown in Table 2. Serum triglycerides, total cholesterol, LDL-Cholesterol, HDL-Cholesterol and fasting plasma glucose levels, BMI, Systolic and diastolic blood pressure values were significantly elevated in the MS group compared to the controls.

**Table 2:** **Major clinical parameters of the patients with metabolic syndrome (MS) and control subjects (means ± SD)**

	**Controls (n = 105)**	**Patients (n = 176)**	**P-value**
**Systolic blood pressure**	11.43 ± 1.31	13.00 ± 1.76	<0.0001
**Diastolic blood pressure**	8.34 ± 6.34	8.43 ± 1.24	<0.0001
**Total cholesterol**	1.90 ± 0.38	2.01 ± 0.46	0.0356
**Triglycerides**	0.96 ± 0.33	1.54 ± 0.69	<0.0001
**LDL-cholesterol**	1.16 ± 0.34	1.28 ± 0.37	0.0063
**HDL-cholesterol**	0.55 ± 0.12	0.48 ± 0.16	0.0001
**Fasting plasma glucose**	0.85 ± 0.09	1.31 ± 0.54	<0.0001
**BMI**	25.34 ± 2.93	31.91 ± 14.09	<0.0001
**Waist circumference**	84.38 ± 10.35	100.47 ± 11.06	<0.0001

HDL: high-density lipoprotein, LDL: low-density lipoprotein, BMI: body mass index.

The results of all analyses of association with disease status are shown in Table 3. The statistical analysis showed that the APOA5 -1131 T > C polymorphism was significantly associated with MS in both Codominant (OR = 10.13; 95% CI:4.65-22.06; p-value < 0.0001) and Dominant (OR = 7.82; 95% CI:3.79-16.14; p-value < 0.0001) models. For the APOA5 c.56C > G polymorphism the statistical analysis showed a significant association in the codominant and dominant models with p-value = 0.035 and 0.032 respectively. On the other hand the data analysis of c.553G > T and c.1259 T > C APOA5 gene polymorphisms showed a no signification association between this SNPs and MS in all genetic models.

**Table 3:** **Association between APOA5 genotypes and Metabolic Syndrome**

**SNP**	**Model**	**Genotype**	**Controls**	**Patients**	**OR** ^**a**^ **(95% CI)**	**P-value**
**−1131 T > C**	Codominant	T/T	65 (61.9%)	48 (28.4%)	1.00	
		T/C	34 (32.4%)	113 (66.9%)	10.13 (4.65-22.06)	<0.0001
		C/C	6 (5.7%)	8 (4.7%)	1.49 (0.37-6.00)	0.575
	Dominant	T/T	65 (61.9%)	48 (28.4%)	1.00	
		T/C-C/C	40 (38.1%)	121 (71.6%)	7.82 (3.79-16.14)	<0.0001
	Recessive	T/T-T/C	99 (94.3%)	161 (95.3%)	1.00	
		C/C	6 (5.7%)	8 (4.7%)	0.52 (0.14-1.99)	0.342
**c.56C > G**	Codominant	C/C	70 (68%)	93 (53.1%)	1.00	
		C/G	28 (27.2%)	64 (36.6%)	2.13 (1.05-4.31)	0.035
		G/G	5 (4.8%)	18 (10.3%)	1.72 (0.48-6.21)	0.407
	Dominant	C/C	70 (68%)	93 (53.1%)	1.00	
		C/G-G/G	33 (32%)	82 (46.9%)	2.07 (1.07-4.03)	0.032
	Recessive	C/C-C/G	98 (95.2%)	157 (89.7%)	1.00	
		G/G	5 (4.8%)	18 (10.3%)	1.27 (0.37-4.36)	0.701
**c.553G > T**	Codominant	G/G	86 (81.9%)	150 (85.2%)	1.00	
		G/T	15 (14.3%)	23 (13.1%)	0.97 (0.38-2.48)	0.943
		T/T	4 (3.8%)	3 (1.7%)	0.27 (0.05-1.59)	0.148
	Dominant	G/G	86 (81.9%)	150 (85.2%)	1.00	
		G/T-T/T	19 (18.1%)	26 (14.8%)	0.73 (0.32-1.71)	0.472
	Recessive	G/G-G/T	101 (96.2%)	173 (98.3%)	1.00	
		T/T	4 (3.8%)	3 (1.7%)	0.26 (0.05-1.53)	0.137
**c.1259 T > C**	Codominant	T/T	53 (51%)	115 (65.7%)	1.00	
		T/C	29 (27.9%)	28 (16%)	0.56 (0.25-1.27)	0.166
		C/C	22 (21.1%)	32 (18.3%)	0.69 (0.32-1.52)	0.365
	Dominant	T/T	53 (51%)	115 (65.7%)	1.00	
		T/C-C/C	51 (49%)	60 (34.3%)	0.63 (0.33-1.17)	0.143
	Recessive	T/T-T/C	82 (78.8%)	143 (81.7%)	1.00	
		C/C	22 (21.1%)	32 (18.3%)	0.80 (0.38-1.68)	0.561

a: ORs were adjusted for sexe, age and body mass index (BMI).

Additionally, we grouped the carriers for the rare allele for all SNPs and compared their frequency against the common allele for all parameters of MS (Table 4). Carriers of the APOA5 -1131C variant were associated with increased fasting glucose (p = 0.0295) and reduced HDL levels (p = 0.0091), compared with noncarriers in MS patients and controls. For the carriers of the APOA5 c.56G variant were associated with increased triglyceride levels (p = 0.0435) and Waist circumference (p = 0.0122), compared with noncarriers in MS patients and controls. Similarly for the APOA5 1259 T > C variant were associated with increased Waist circumference (p = 0.0463).

**Table 4:** **Comparisons of clinical and biochemical parameters of the study participants according to the APOA5 -1131 T > C, c.56C > G, c.553G > T and c.1259 T > C genotypes**

	**APOA5 -1131 T > C**			**APOA5 c.56C > G**			**APOA5 c.553G > T**			**APOA5 c.1259 T > C**		
	**TT**	**TC/CC**	**P**	**CC**	**CG/GG**	**P**	**GG**	**GT/TT**	**P**	**TT**	**TC/CC**	**P**
**Systolic blood pressure**	12.08 ± 1.63	12.60 ± 1.81	0.0162	12.37 ± 1.87	12.50 ± 1.65	0.5348	12.43 ± 1.74	12.37 ± 1.95	0.8487	12.48 ± 1.77	12.34 ± 1.79	0.5173
**Diastolic blood pressure**	8.75 ± 6.11	8.14 ± 1.21	0.7573	8.18 ± 1.21	8.71 ± 6.06	0.9794	8.47 ± 4.32	7.98 ± 1.16	0.1979	8.59 ± 5.07	8.10 ± 1.16	0.3322
**Total cholesterol**	1.97 ± 0.39	1.96 ± 0.46	0.8041	1.98 ± 0.45	1.95 ± 0.42	0.5260	1.98 ± 0.42	1.94 ± 0.50	0.5905	1.98 ± 0.43	1.95 ± 0.44	0.6621
**Triglycerides**	1.23 ± 0.58	1.37 ± 0.68	0.0892	1.24 ± 0.55	1.44 ± 0.75	0.0435	1.31 ± 0.63	1.41 ± 0.70	0.3722	1.34 ± 0.68	1.29 ± 0.59	0.8394
**LDL-cholesterol**	1.21 ± 0.33	1.26 ± 0.39	0.3161	1.23 ± 0.38	1.24 ± 0.35	0.9080	1.24 ± 0.37	1.23 ± 0.38	0.9020	1.24 ± 0.35	1.22 ± 0.39	0.6142
**HDL-cholesterol**	0.54 ± 0.15	0.49 ± 0.15	0.0091	0.50 ± 0.14	0.51 ± 0.17	0.5918	0.51 ± 0.15	0.49 ± 0.14	0.5002	0.51 ± 0.16	0.50 ± 0.13	0.4028
**Fasting plasma glucose**	1.03 ± 0.34	1.20 ± 0.52	0.0295	1.11 ± 0.46	1.18 ± 0.51	0.3953	1.13 ± 0.47	1.17 ± 0.52	0.8328	1.16 ± 0.49	1.11 ± 0.46	0.2787
**BMI**	30.06 ± 17.46	29.04 ± 5.06	0.2244	29.44 ± 14.81	29.48 ± 5.04	0.0617	29.72 ± 12.59	28.08 ± 5.05	0.4439	29.27 ± 5.35	29.67 ± 17.47	0.0639
**Waist circumference**	93.53 ± 13.00	94.95 ± 13.77	0.3919	92.75 ± 13.26	96.81 ± 13.18	0.0122	94.68 ± 13.09	93.29 ± 14.47	0.5213	95.73 ± 13.50	92.48 ± 12.92	0.0463

HDL: high-density lipoprotein, LDL: low-density lipoprotein, BMI: body mass index.

The PLINK program was then used to estimate haplotype frequencies and identify haplotype association with disease (Table 5). The study data show that haplotype-based analysis appears to confirm the SNP analysis with respect to the association of -1131C and 56G alleles with MS. Two haplotypes showed significant association with MS. Haplotypes CCGT (OR = 3.223; 95% CI:1.43-7.25; p = 0.00278) and CGGT (OR = 8.234; 95% CI:1.6-42.5; p = 0.00534) confer susceptibility to MS,

**Table 5:** **Association analysis of the APOA5 haplotypes**

**HAPLOTYPE**	**Frequency**		**OR** ^**a**^ **(95%CI)**	**P-value**
	**Patients**	**Controls**		
**TCGT**	0.3941	0.4749	0.6667 (0.406; 1.09 )	0.106
**CCGT**	0.2343	0.1485	3.223 (1.43; 7.25 )	0.00278
**TCGC**	0.1489	0.2295	0.6331 (0.352; 1.14 )	0.122
**TGGT**	0.1196	0.1129	1.013 (0.418; 2.45 )	0.978
**CGGT**	0.1032	0.03414	8.234 (1.6; 42.5 )	0.00534

a: ORs were adjusted for sexe, age and body mass index (BMI).

The data for the haplotype association are shown in Table 6. We found only nominal association with BMI (p = 0.0269) and waist circumference (p = 0.0268) for haplotype CGGT.

**Table 6:** **Association between APOA5 haplotypes with clinical and biochemical parameters**

	**Haplotype TCGT**			**Haplotype CCGT**			**Haplotype CGGT**		
	**Carriers**	**Noncarriers**	**P-value**	**carriers**	**Noncarriers**	**P-value**	**carriers**	**Noncarriers**	**P-value**
**Systolic blood pressure**	12.47 ± 1.72	12.34 ± 1.86	0.5482	12.46 ± 1.89	12.40 ± 1.73	0.7790	12.55 ± 1.49	12.39 ± 1.82	0.5936
**Diastolic blood pressure**	8.62 ± 5.07	8.06 ± 1.14	0.2070	8.04 ± 1.24	8.54 ± 4.66	0.3589	8.07 ± 1.10	8.45 ± 4.30	0.5528
**Total cholesterol**	1.98 ± 0.40	1.95 ± 0.49	0.5102	1.96 ± 0.48	1.97 ± 0.42	0.7929	2.04 ± 0.44	1.96 ± 0.43	0.2623
**Triglycerides**	1.34 ± 0.67	1.30 ± 0.60	0.9167	1.27 ± 0.62	1.34 ± 0.65	0.2034	1.43 ± 0.82	1.30 ± 0.61	0.4413
**LDL-cholesterol**	1.23 ± 0.34	1.25 ± 0.40	0.6883	1.25 ± 0.40	1.23 ± 0.35	0.6162	1.34 ± 0.42	1.22 ± 0.36	0.0526
**HDL-cholesterol**	0.51 ± 0.15	0.49 ± 0.15	0.2427	0.48 ± 0.14	0.52 ± 0.16	0.0868	0.51 ± 0.18	0.50 ± 0.14	0.7061
**Fasting plasma glucose**	1.17 ± 0.50	1.10 ± 0.46	0.0906	1.18 ± 0.51	1.12 ± −0.47	0.6943	1.17 ± 0.50	1.13 ± 0.48	0.6330
**BMI**	29.09 ± 5.20	30.00 ± 17.40	0.4779	28.34 ± 5.01	29.91 ± 13.51	0.4044	30.39 ± 5.71	29.29 ± 12.48	0.0269
**Waist circumference**	94.43 ± 11.88	94.50 ± 15.24	0.9685	92.64 ± 13.12	95.19 ± 13.34	0.1459	98.64 ± 17.13	93.72 ± 12.41	0.0268

HDL: high-density lipoprotein, LDL: low-density lipoprotein, BMI: body mass index.

## Discussion

During the last years, the rapid increase in MS prevalence in industrialized countries coupled with its devastating complications on human health, mainly because of a higher risk for developing cardiovascular disease, a leading cause of death [4]. MS attracted a substantial interest in deciphering the major genetic factors that contribute to its pathogenic mechanism [27].

The role of APOA5 polymorphisms (−1131 T > C, c.56C > G, c.553G > T and c.1259 T > C) and their involvement in various diseases have been studied extensively throughout the world in different populations. To our knowledge, this is the first study to test the association between APOA5 polymorphisms and MS in the Moroccan population.

We found a strong association between -1131 T > C polymorphism and MS in both dominant and codominant models. This association was previously reported by Maasz et al. in European subjects [25]. A meta-analysis showed that the -1131 T > C polymorphism was significantly associated with the MS risk in Asian populations [28]. Obese adolescents with the -1131C allele had an increased risk for the development of metabolic syndrome [29].

The c.56C > G (p.Ser19Trp) polymorphism, resulting in a substitution of hydrophilic serine to hydrophobic tryptophan, showed an association with the MS in both domimant and codominant models. On the other hand, the statistical analysis of genotype distributions revealed no association between c.553G > T and c.1259 T > C APOA5 polymorphisms and MS. In Japanese population, the c.553G > T variant significantly associated with hypertriglyceridemia [30].

Several SNPs in the APOA5 locus have been identified in humans, four of them, the -1131 T > C, IVS3 + 476G > A, 1259 T > C and c.56C > G represent the most common variants. As these genetic natural variants have an effect on the transcriptional activity of apoa5 protein, some alleles have been reported to be associated with high plasma TG levels. The association between -1131C, IVS3 + 476A and 1259C alleles and high TG levels was confirmed in patients with the metabolic syndrome and in healthy controls [26]. The -1131C allele was significantly associated increased serum triglyceride levels in Korean subjects [31]. The modified triglyceride metabolism may be involved in the abnormal accumulation of fat in the vascular endothelial cells, and in pathological conditions, it may also be involved in the formation of atheroma plaques which are associated with disease processes leading to the appearance ischemic vascular diseases. The -1131 T > C polymorphism have been reported to confer risk of coronary heart disease and ischemic stroke in many studies in different populations [10][32].

In this study we found that carriers of the APOA5 -1131C had increased systolic blood pressure, fasting blood glucose and reduced levels of HDL. Similarly, other studies reported a significant association between low HDL and this variant in Caucasians [33,34] and Asian populations [30]. In nonobese Korean men, no significant association was found between APOA5 -1131C variant and fasting plasma glucose [15].

In addition, carriers of the APOA5 c.56G allele had an increased TG levels and waist circumference compared to patients and controls non-carriers of this variant. A similar result was reported in several studies [35,36]. Moreover, the APOA5 c.1259 T > C variant was associated with higher waist circumference.

Cross-sectional studies have shown that several polymorphisms in or near the APOA5-A4-C3-A1 gene, are associated with triglycerides. In a performed in 199 subjects from North Iranian population, two APOA5 gene polymorphisms, −1131 T > C and c.56C > G were significantly associated with Triglycerides and Waist-to-Hip Ratio, respectively [37]. Among patients with clinically manifest vascular disease, the polymorphism rs964184 in APOA5-A4-C3-A1 gene cluster was associated with higher plasma triglycerides concentrations [38].

Our results show no association between APOA5 c.553G > T polymorphism and clinical and biochemical parameters. In Asian populations, the minor allele of the APOA5 c.553G > T polymorphism was associated with higher TG levels [19,39]. This functional variant occurs in the coding region of APOA5 gene and causes a substitution of a cysteine by a glycine residue, was suggested as prognostic indicators for hypertriglyceridemia risk in Chinese [17]. Another study suggests that the APOA5 c.553G > T polymorphism has no influence directly in the expression of the APOA5 [39].

In this study, we performed haplotype analysis to evaluate the susceptibility to MS. We observed that the CCGT (APOA5*2) haplotype determined by the combination of alleles -1131C, c.56C, c.553G and c.1259 T is associated with the MS (OR = 3.223, 95% CI:1.43- 7.25, p = 0.00278), but no association was found with biochemical parameters. However, Kisfali et al. found an association between the haplotype APOA5*2 and high plasma TG levels among patients with MS and controls [26], the same result was found in other studies in normal subjects [13,40].

We found a second association between the CGGT (APOA5*5) haplotype and increased risk of MS (OR = 8.234, 95%CI: 1.6-42.5, p = 0.0053) and higher BMI (p = 0.0269) and waist circumference (p = 0.0268). Among the Hungarian population, the APOA5*2 haplotype (the combination of -1131 T > C, IVS3 + 476G > A and 1259 T > C SNPs) confers risk for the development of MS (OR = 2.880; 95% CI: 1.567-5.292; p = 0.001), and a novel haplotype APOA5*5 (1259C allele alone) with a protective effect against MS was identified [26]. In 2005, Talmud et al., showed that the polymorphism c.56C > G which characterize the haplotype APOA5*3, reduced about 50% secretion of the protein apoAV in a cellular model of translocation of the protein across the endoplasmic reticulum, which could explain the increased plasma TG in carriers of the APOA5*3 haplotype [41]. Several studies performed association analysis using haplotypes constructed with SNPs located in different genes including APOA5. A particular haplotype determined by SNPs in APOA5 and ZNF259 genes, showed significant association with TG:HDL-C ratio and the risk of MS in both genders with marked effects in women [42]. Analysis of constructed haplotypes with SNPs in MTHFR, APOA5 and APOC3 genes, showed a highly significant association between one haplotype and MS in the Greek Population [43]. Gene-gene interaction suggested that polymorphisms in APOA5 and BTN2A1 genes may have synergistic effects on the development of MS in Japanese individuals [44].

There are some limitations in this work, mainly the low sample size. Replicating our findings in larger Moroccan cohorts from different ethnic groups and geographic regions, could overcome these limitations and provide sufficient statistical power to reach clear conclusion on the role of APOA5 polymorphisms in metabolic syndrome susceptibility. Moreover, only four SNPs in APOA5 gene were investigated in this study, we cannot exclude the possibility that other SNPs or particular haplotypes may have a significant impact on genetic susceptibility to metabolic syndrome. The target of our future studies is investing the association between high number of SNPs per gene and genetic susceptibility to metabolic syndrome, in large cohorts from Moroccan population.

In conclusion, we found that the APOA5 (−1131 T > C and c.56C > G) variants and haplotypes (CCGT and CGGT) were significantly associated with susceptibility to MS in Moroccan patients. This finding suggests that APOA5 polymorphisms and haplotypes could be used as predictive indicators for MS in Moroccan population. In addition, prompt diagnosis and treatment of individuals with multiple risk factors, and will contribute substantially to prevention by adopting a healthy lifestyle and reducing future complications.
